# Cybervictimization and emotional symptoms in adolescents: mediating role of psychological flexibility versus inflexibility

**DOI:** 10.3389/fpsyg.2024.1505422

**Published:** 2025-01-15

**Authors:** María del Mar Molero Jurado, África Martos Martínez, María del Carmen Pérez-Fuentes, Rosa María del Pino Salvador, José Jesús Gázquez Linares

**Affiliations:** ^1^Department of Psychology, Faculty of Psychology, University of Almería, Almería, Spain; ^2^Department of Psychology, Universidad Autónoma de Chile, Providencia, Chile

**Keywords:** psychological flexibility, cyberbullying, adolescence, psychological inflexibility, cybervictimization, emotional symptoms

## Abstract

**Background:**

Cyberbullying significantly affects adolescents, increasing the risk of negative emotional symptoms. This study explores how psychological flexibility and inflexibility may mediate this relationship, focusing on adolescent mental health in the context of cyberbullying.

**Methods:**

A sample of 2,171 secondary school students, aged 11–18 years, was used to assess cyberbullying, emotional symptoms, and levels of psychological flexibility and inflexibility.

**Results:**

Cybervictimization showed a direct relationship with emotional symptoms and psychological inflexibility. Psychological flexibility was negatively associated with cybervictimization and positively associated with mental health, acting as a protective mediator against the association of cyberbullying.

**Conclusion:**

Psychological flexibility emerges as a key factor in reducing the negative association of cybervictimization in adolescents. These findings highlight the importance of developing strategies to improve psychological flexibility in young people as a means to strengthen their resilience in the face of cyberbullying and its emotional consequences.

## 1 Introduction

Increasing exposure to virtual media among young people can pose serious problems, as among other adverse events (Arrivillaga et al., 2022; Herruzo et al., 2023; Hidalgo, 2022), it increases the risk of becoming a cybervictim (Aizenkot, 2022). Cybervictimization refers to intentionally acting to annoy or inflict harm on others, making use of the electronic context, including text messages, online groups and games, emails, calls and social networks (Viau et al., 2020). These behaviors are common and present in virtually all parts of the world (Ruiz-Esteban et al., 2022), generating great concern (Molero et al., 2022). As far as Spain is concerned, cybervictimization affects 7.4% of adolescents from majority groups. But this figure rises to more than 20% in the case of groups such as immigrants or sexual minorities (Zych and Llorent, 2021).

Co-construction theory (Subrahmanyam et al., 2008) suggests that the adolescent’s social world is similar both online and face-to-face. Thus, prosocial adolescents would also be prosocial in their online experiences, while young people who exhibit violent behaviors would tend to do so in cyberspace as well (Ehrenreich et al., 2021). Similarly, as with traditional bullying, being a victim of cyberbullying is linked to negative consequences for health and psychological wellbeing (García-Vázquez et al., 2022; Pérez-Fuentes et al., 2016). Although research on cyberbullying has advanced in recent years, it remains a major challenge to identify ways to protect young people from the lasting harms of cyberbullying (Quintana-Orts et al., 2021a). Psychological inflexibility has been established as a significant mediator of the relationship between early adverse experiences and the onset of depression and anxiety (Makriyianis et al., 2019). In this study, we explored the role of psychological flexibility and inflexibility to determine whether they mediate the relationship between cybervictimization and negative emotional symptoms. Psychological flexibility refers to the ability to appreciate our thoughts and emotions without judgment, preventing them from affecting our behavior. Psychological inflexibility is the domination of an individual’s behavior by a cognitive and/or emotional pattern that is dysfunctional and does not attend to our life choices (Hayes et al., 2006). Young people who have been victims of cyberbullying may be less able to push away their negative thoughts, being more likely to develop mental health problems. In contrast, if adolescents are psychologically flexible, they will experience their emotions and thoughts without having their goals and values altered, i.e., without it affecting their psychological wellbeing. The World Health Organization (2022) defines mental health as a state of psychological wellbeing that enables people to cope with everyday stress and develop their individual potential. Mental health is more than the absence of disorders, and the problems that compromise it can range from mental disorders and disability, to mental and emotional states of distress that undermine psychological wellbeing. Among the circumstances that increase the risk of mental health conditions is bullying.

Cybervictimization refers to being a target of aggression through electronic devices, mainly mobile phones and the internet. It can take different forms. Visual cybervictimization involves the dissemination of offensive or hurtful images or videos. Written-verbal cybervictimization involves receiving threatening or offensive phone calls, messages or written comments. Online exclusion refers to being rejected from a group, usually on social networks or instant messaging. Finally, we talk about cybervictimization by impersonation when someone mocks the victim or creates problems for them by impersonating them (Álvarez-García et al., 2017).

Cyberbullying does not cause physical harm and therefore its consequences are less visible, although particularly severe and disturbing (Lindfors et al., 2012). Being cyberbullied has been linked to negative consequences on psychological and emotional adjustment in the short- and long-term (Zych et al., 2015). It is highly associated with emotional impact, such as negative feelings and difficulty regulating emotions (Quintana-Orts et al., 2021b; Rodríguez-Álvarez et al., 2021). It is therefore not surprising that it is related to depression (Viau et al., 2020; Franzen et al., 2024), anxiety and stress (Molero et al., 2023). It has even been identified as a risk factor for its development (Hong et al., 2015), especially among girls (Fredrick and Demaray, 2018).

Other mental health problems at higher risk of developing among young people who experience cyberbullying, compared to non-victims, are post-traumatic stress disorder (PTSD), substance abuse, gambling addiction (Zhu et al., 2021), and self-harming behaviors (Zhao et al., 2022). Increased risk of experiencing sadness, anxiety, or psychosomatic problems has also been reported (Li et al., 2022). In terms of psychosocial adjustment, cybervictimization increases suicidal ideation, both directly and indirectly through increased feelings of loneliness, stress, psychological distress, and depressive symptoms (Iranzo et al., 2019). In this sense, victimization in adolescence has been linked to increased social anxiety (Camacho et al., 2022).

Gender differences in the mental health effects of cybervictimization have been reported. Specifically, while women who are cyberbullied experience more emotional problems, for men cybervictimization is linked more to behavioral problems (Kim et al., 2018). These associations between cybervictimization and mental health problems are stronger than in the case of traditional bullying. Thus, victims of cyberbullying are affected in their quality of life, but not only on a mental level. In addition to poorer psychological wellbeing and mood, students who are cyberbullied show poorer relationships with parents and family members, poorer physical wellbeing, social and self-acceptance, and perceive their school environment as less affable than young people who perpetrate cyberbullying or who are not involved in cyberbullying (González-Cabrera et al., 2018).

Young people’s response to online aggression can determine whether they recover quickly or their mental health is impaired (Paris et al., 2022). Psychological flexibility is a construct derived from Acceptance and Commitment Therapy (ACT; Hayes et al., 2011), which allows one to experience one’s thoughts and emotions in a non-judgmental way and to act in accordance with one’s values and goals. In contrast, psychological inflexibility is the domination of an individual’s behavior by their feelings and thoughts, without regard for their goals (Bond et al., 2011) or the demands they face (Ferradás et al., 2021). It is the tendency to persevere in a cognitive, emotional or behavioral pattern that is no longer functional, due to a lack of sensitivity to the demands of the context (Giommi et al., 2023). So it is a matter of accepting or not accepting difficult emotions and thoughts, while remaining committed to taking actions consistent with one’s chosen values (Hayes et al., 2006). Although the two variables seem contrary, they are not part of a continuum, so they should be measured independently (Rolffs et al., 2016).

Psychological flexibility has been shown to mitigate victimization outcomes among young people. For example, Duarte and Pinto-Gouveia (2016) found that psychological flexibility about body image reduced deviant eating behaviors and promoted an appropriate body mass index among youth who had experienced teasing and bullying due to weight. And in terms of cybervictimization, Zhao et al. (2022) found that difficulty regulating intense emotions acted as a negative mediator for the occurrence of self-injurious behaviors in adolescents, while dispositional mindfulness (i.e., the innate tendency to focus on the present moment, without judging or reacting to internal experience) mediated the reduction of these behaviors.

As for psychological inflexibility, its involvement in emotional distress has been reported (Fernández-Rodríguez et al., 2022). Young people who show higher levels of this variable have more depressive symptoms and lower life satisfaction (Lappalainen et al., 2021). Its mediating role between adverse childhood events and depression has also been identified (Zhao et al., 2022). Little is known about its role in victims of cyberbullying. However, it has been analyzed in other groups exposed to distressing situations. For example, in adolescents belonging to sexual minorities, mental rigidity has been identified as a moderator of the relationship between stress and substance abuse, as well as being directly linked to suicidal tendencies (Weeks et al., 2020). This highlights the strong impact on mental health of this variable, which fosters persistence and impedes change and actions aimed at achieving a valued life (Hayes et al., 2006). Furthermore, Quintana-Orts et al. (2021a) indicate that victim responses to cyberbullying such as avoiding thinking about it, waiting for it to stop, is often interpreted as weakness on the part of others, encouraging further abuse, which could lead to further psychological distress.

The purpose of this article is to expand knowledge about how cybervictimization is related to negative emotional symptoms by examining the role of psychological flexibility and inflexibility as possible mediators. To this end, we first aim to investigate the relationships between cybervictimization, emotional symptoms, flexibility, and psychological inflexibility. On the other hand, we propose to analyze the mediating role of psychological flexibility and inflexibility in the association of cybervictimization on the emotional symptoms of adolescents. We hypothesize that psychological inflexibility will make the development of depression, anxiety, and stress among young people more likely. Conversely, flexibility will make it less likely.

## 2 Materials and methods

### 2.1 Participants

This quantitative study was conducted using a descriptive cross-sectional design. Initially, the selection of the centers was carried out by means of random sampling by clusters, according to the different geographical and regional areas of the Province of Almeria. In this way, two centers were selected from the four areas of the province (Inland, Metropolitan Region of Almeria, Levante Almeriense, and Poniente Almeriense). As a result, eight centers in Almeria were finally contacted. In a second stage, the participants were selected using cluster sampling. The initial sample consisted of 2,241 adolescents who agreed to participate voluntarily in the research. Of these, 70 were discarded because they did not complete the entire battery of questionnaires. Thus, the sample finally consisted of 2,171 secondary school students (the response rate was 96.88%). Age ranged from 11 to 18 years, with a mean of 13.84 years (SD = 1.46). In terms of gender, 50.53% (*n* = 1,097) were male and 49.47% (*n* = 1,074) were female, with a mean of 13.88 (SD = 1.45) and 13.81 (SD = 1.46) years, respectively. All participants were enrolled in various public secondary schools in the province of Almería (Spain), which offer three educational pathways: compulsory secondary education, high school, and vocational training. In Spain, compulsory secondary education consists of four academic years (ages 12–16), which students must complete to fulfill legal education requirements. The distribution in terms of the grade were the following: 25.51% (*n* = 554) were of first year of compulsory education, 22.98% (*n* = 499) 2nd year of compulsory education, 23.35% (*n* = 507) 3rd year of compulsory education, 19.94 (*n* = 433) 4th year of compulsory education, 4.33% (*n* = 94) 1st year high school, 3.27% (*n* = 71) 2nd year high school, and 0.59% (*n* = 13) vocational training.

### 2.2 Measures

The Cybervictimization Questionnaire (CYVIC; Álvarez-García et al., 2017) for adolescents assesses the extent to which the informant has been a victim of aggression in the last 3 months via mobile phone or internet. It consists of 19 items with 4 Likert-type alternatives (from “never” to “always”) from which 4 scales are obtained: impersonation (online simulation of the victim’s profile; e.g., “Someone who has got my password has sent annoying messages to someone I know, as if it was me, to get me into trouble.”), visual-sexual cybervictimization (involving the recording or photographing and online dissemination of compromising and/or humiliating images; e.g., “Someone has posted real compromising photos or videos of me on the Internet without my permission, to hurt me or make fun of me.”), verbal-written cybervictimization (related to receiving anonymous annoying or threatening calls, as well as hurtful and frightening comments via the internet; e.g., “Someone has mocked me with offensive or insulting comments on social media.”), and online exclusion (being deliberately left out of an online group; e.g., “They agree to ignore me on social networks.”). The scale showed adequate reliability and validity indices (Álvarez-García et al., 2017). The reliability found in this study was: α = 0.662 for Impersonation, α = 0.662 for Visual-Sexual Cybervictimization, α = 0.812 for Written-Verbal Cybervictimization, and α = 0.704 for Online Exclusion.

Spanish version of Depression Anxiety and Stress Scale (DASS-21; Ruiz et al., 2017) describes negative emotional states based on 21 items that are grouped around 3 scales: depression (e.g., “I felt there was nothing to look forward to.”), anxiety (e.g., “I felt my heart beating even though I had not made any physical effort.”), and stress (e.g., “He tendido a sentirme enfadado con facilidad”). Responses follow a 4-point Likert-type scale, based on the frequency with which they experienced each item during the previous week, and where 0 equals “not at all applicable to me” and 3 “totally applicable to me or most of the time.” This instrument showed good psychometric properties (Ruiz et al., 2017). Reliability for the scales in this study was as follows: Depression α = 0.884, Anxiety α = 0.832, and Stress α = 0.786.

Work-related Acceptance and Action Questionnaire (WAAQ; Bond et al., 2013; Ruiz and Odriozola-González, 2014). It consists of 7 items that assess psychological flexibility in the academic domain (e.g., “the willingness to perform actions to achieve a goal while experiencing situations that cause discomfort”). The items are answered on a 7-point Likert-type scale (from “never true” to “always true”). The instrument showed adequate psychometric properties (Ruiz and Odriozola-González, 2014). In this case, a reliability of α = 0.844 was obtained.

Spanish version of Acceptance and Action Questionnaire-II (AAQ-II; Ruiz et al., 2013). This instrument assesses psychological inflexibility based on 7 items answered on a 7-point Likert-type scale (from 1 “never true” to 7 “always true”). The items reflect the difficulty in experiencing unwanted emotions and thoughts, as well as the inability to achieve psychological wellbeing because of them (e.g., “My worries get in the way of what I want to achieve.”). In the original study (Ruiz et al., 2013) adequate psychometric data were found. The reliability obtained was α = 0.877.

### 2.3 Procedure

Initially, school principals were contacted to inform them of the objectives of the study and to guarantee the confidentiality of the data. Data collection was carried out by two members of the team who went to the schools to administer the questionnaires. The researchers explained the instructions and ethical considerations to the students. In all cases, in compliance with ethical research standards, all participants accepted voluntary participation and had written consent from parents/guardians at the school for their participation. The study was approved by the Bioethics Committee of the University of Almería (Ref: UALBIO2021/022).

### 2.4 Statistical analysis

As preliminary analyses, the correlation matrix between the variables included in the study is presented, as well as the mean scores and standard deviation. In addition, the existence of statistically significant differences according to gender was examined, with a comparative analysis of the mean scores of all the variables involved in the study. For this purpose, Welch’s (1947) *t*-test and Cohen’s (1988) *d* coefficient for effect size (0.20, 0.50, and 0.80 to interpret observed effect sizes as small, medium, or large, respectively) were applied. To estimate the reliability of the instruments, data on Cronbach’s (1951) alpha coefficient are provided.

On the other hand, in order to test the position of psychological flexibility and inflexibility in the model, SEM analysis was applied. Specifically, a latent mediation model was computed using the Maximum Likelihood (ML) estimation method, specifying two pathways of association of cybervictimization with the presence of emotional symptoms: a direct effect and an indirect effect through psychological flexibility (model 1) and psychological inflexibility (model 2). For this purpose, the lavaan package (Rosseel, 2012), integrated in JASP version 0.16.3 (JASP Team, 2022), is used. The following indices were used to assess model fit: the Chi-square ratio/degrees of freedom (χ^2^/df), which is considered optimal with values <3 (Iacobucci, 2010; Kline, 2005) and acceptable <5 (Bentler, 1989); the CFI, TLI, and GFI indices, which according to Hu and Bentler (1999) should provide values >0.95 to be considered an optimal fit and >0.90 for an acceptable fit; and RMSEA, which considers values <0.06 optimal and <0.08 or very close values, acceptable.

## 3 Results

### 3.1 Preliminary analyses: descriptive and correlations

First, Table 1 shows that Psychological Flexibility has negative associations with all four types of cybervictimization: Impersonation (*r* = 0.11, 95% CI = 0.15, 0.02), Visual Sexual Cybervictimization (*r* = 0.08, 95% CI = 0.13, 0.04), Written-Verbal Cybervictimization (*r* = 0.16, 95% CI = 0.20, 0.12), and Online Exclusion (*r* = 0.11, 95% CI = 0.15, 0.07). Similarly, Psychological Flexibility correlates negatively with Depression (*r* = 0.32, 95% CI = 0.36, 0.28), Anxiety (*r* = 0.26, 95% CI = 0.30, 0.22), and Stress (*r* = 0.23, 95% CI = 0.27, 0.19).

**TABLE 1:** Descriptive statistics and correlation analysis of the study variables.

Variable (*M* ± SD)	Psychological flexibility (27 ± 9.74)	1	2	3	4	5	6	7
1. Impersonation (3.47 ± 9.74)	0.113	–						
2. Visual sexual cybervictimization (3.49 ± 1.20)	0.088	0.582	–					
3. Written-verbal cybervictimization (8.42 ± 3.34)	0.166	0.615	0.553	–				
4. Online exclusion (3.89 ± 1.62)	0.113	0.548	0.547	0.636	–			
5. Depression (8.09 ± 5.85)	0.327	0.174	0.150	0.316	0.275	–		
6. Anxiety (7.50 ± 5.42)	0.269	0.207	0.187	0.349	0.312	0.754	–	
7. Stress (8.95 ± 5.02)	0.235	0.156	0.122	0.281	0.252	0.706	0.740	–
Psychological inflexibility (38.12 ± 14.21)	0.422	0.177	0.141	0.273	0.249	0.621	0.536	0.533

All associations were significant at *p* < 0.001.

In contrast, in the case of Psychological Inflexibility, there are positive associations with the different modalities of cybervictimization: Impersonation (*r* = 0.17, 95% CI = 0.13, 0.21), Visual Sexual Cybervictimization (*r* = 0.14, 95% CI = 0.09, 0.18), Written-Verbal Cybervictimization (*r* = 0.27, 95% CI = 0.23, 0.31), and Online Exclusion (*r* = 0.24, 95% CI = 0.20, 0.28).

Finally, as can be seen in the correlation matrix, the associations between types of cybervictimization and emotional symptoms are positive for all cases.

**FIGURE 1:**
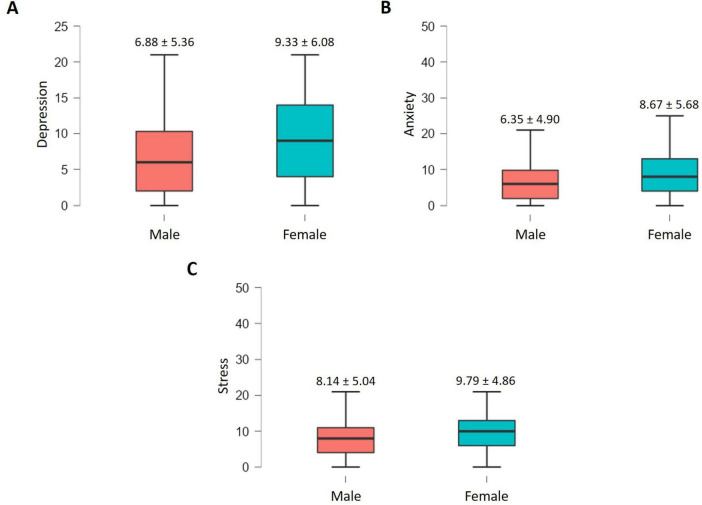
**(A)** Depression by sex; **(B)** anxiety by sex; **(C)** stress by sex.

On the other hand, Figures 1A–C show graphically the differences in the mean scores obtained for each of the emotional symptoms, according to gender. As shown, female have significantly higher mean scores for depression (*t* = 9.92, *p* < 0.001, *d* = 0.42), anxiety (*t* = 10.16, *p* < 0.001, *d* = 0.43), and stress (*t* = 7.76, *p* < 0.001, *d* = 0.33), compared to males.

**FIGURE 2:**
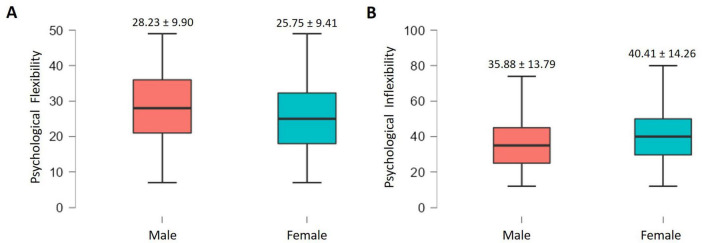
**(A)** Psychological flexibility by sex; **(B)** psychological inflexibility by sex.

In relation to gender differences in psychological flexibility and inflexibility, mean scores are shown in Figures 2A, B, respectively. In the male sex, mean scores in psychological flexibility are significantly higher (*t* = 5.97, *p* < 0.001, *d* = 0.25) than in the female sex. While it is the latter who differ significantly in the scores obtained in Psychological Inflexibility (*t* = 7.51, *p* < 0.001, *d* = 0.32), compared to the male.

### 3.2 Cybervictimization and emotional symptoms: flexibility or not flexibility

**FIGURE 3:**
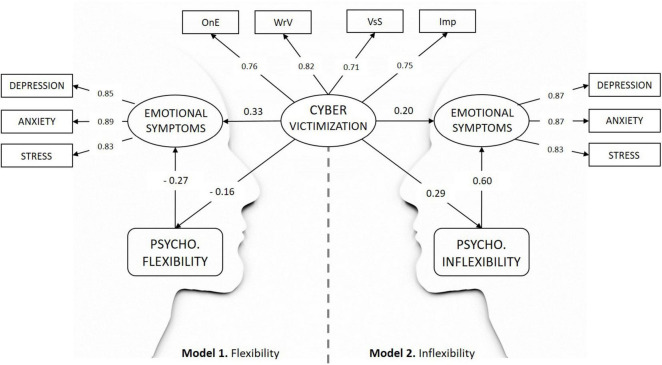
Cybervictimization and emotional symptoms relations (model 1: psychological flexibility as mediator, and model 2: psychological inflexibility as mediator). OnE, online exclusion; WrV, written-verbal cybervictimization; VsS, visual sexual cybervictimization; Imp, impersonation. All associations were significant (*p* < 0.001). All standardized estimates.

Model 1 (Figure 3, left side) showed an acceptable fit, as indicated by the values obtained (Table 2). The relationships established between the latent variables of the model are as follows: cybervictimization is negatively related to psychological flexibility and positively related to the presence of emotional symptoms. On the other hand, the direct relationship between psychological flexibility and the latent variable of emotional symptoms (constructed from the dimensions of the DASS-21) has a negative sign.

**TABLE 2:** Regression coefficients and models fit indices.

	Predictor → outcome	Estimate	SE	*z*	*p*	95% CI		Std. estimate
						**Lower**	**Upper**	
Model 1	CV → ES	1.84	0.13	13.90	<0.001	1.57	2.09	0.33
	PsychoFlex → ES	0.14	0.01	12.61	<0.001	0.15	0.11	0.27
	CV → PsychoFlex	1.77	0.25	7.09	<0.001	2.25	1.27	0.16
Model 2	CV → ES	1.14	0.11	10.04	<0.001	0.92	1.36	0.20
	PsychoInflex → ES	0.21	0.01	30.91	<0.001	0.20	0.22	0.60
	CV → PsychoInflex	4.49	0.36	12.37	<0.001	3.78	5.20	0.29
Models fit indices		Chi-square test			RMSEA (90% CI)	IFC	TLI	GFI
		χ^2^	df	*p*				
Model 1		188.46	18	<0.001	0.066 (0.058, 0.075)	0.978	0.967	0.997
Model 2		231.03	18	<0.001	0.074 (0.066, 0.082)	0.976	0.962	0.994

SE, standard error; CI, confidence intervals; Std. estimate, standardized estimate; RMSEA, root mean square error of approximation; CFI, comparative fit index; TLI, Tucker-Lewis index; GFI, goodness of fit index; CV, cybervictimization; ES, emotional symptoms; PsychoFlex, psychological flexibility; PsychoInflex, psychological inflexibility.

On the other hand, in model 1, taking into account the total effect of cybervictimization on the presence of emotional symptoms (Estimate = 2.08; SE = 0.14; *z* = 15.19; *p* < 0.001; 95% CI 1.812, 2.349; Std. estimate = 0.38), and taking into account the magnitude of the indirect effect (Estimate = 0.24; SE = 0.04; *z* = 6.33; *p* < 0.001; 95% CI 0.168, 0.318; Std. estimate = 0.04), we can conclude that the proportion (indirect/total = 0.11) of this effect mediated by psychological flexibility is between 0.081 and 0.152.

Model 2 (Figure 3, right side) showed an acceptable fit, as indicated by the values obtained (Table 2). The relationships established between the latent variables of the model are as follows: cybervictimization is positively related to psychological inflexibility and also to the presence of emotional symptoms. The direct relationship between psychological inflexibility and the presence of emotional symptoms is positive.

Finally, in model 2, taking into account the total effect of cybervictimization on the presence of emotional symptoms (Estimate = 2.11; SE = 0.14; *z* = 15.16; *p* < 0.001; 95% CI 1.839, 2.385; Std. estimate = 0.37), and taking into account the magnitude of the indirect effect (Estimate = 0.97; SE = 0.08; *z* = 11.64; *p* < 0.001; 95% CI 0.804, 1.130; Std. estimate = 0.17), we can estimate that the proportion (indirect/total = 0.46) of this effect mediated by psychological inflexibility is between 0.393 and 0.523.

## 4 Discussion

The use of interactive digital devices is rapidly increasing among adolescents. More specifically, social networks are a powerful tool very present in the daily lives of minors and their use is related to important aspects of psychosocial development (Mestre-Bach et al., 2022; Ehrenreich et al., 2021). However, the characteristics of cyberspace establish it as an ideal setting for the emergence of cyberbullying (Ferreira and Deslandes, 2018). The results of this study indicated the existence of positive relationships between cybervictimization and the symptoms of depression, anxiety, and stress. This supports previous findings pointing to the relationship between being a victim of cyberbullying and alterations in psychological wellbeing (Méndez et al., 2021; Zych et al., 2015). Furthermore, cyberbullying showed a negative association with psychological flexibility and a positive association with inflexibility. This points to the difficulty in accepting emotions and that these do not dominate behavior among cybervictims. Specifically, adolescents who are cybervictims may have difficulty disengaging from dysfunctional emotional and thought patterns, letting these guide their behavior and moving away from achieving valuable goals in their lives. There is therefore a relationship between receiving cyber violence and acting under emotional and/or cognitive states that may not align with what they really want for themselves.

Another of the results of this study pointed to the existence of differences in emotional symptoms according to gender, with girls having higher scores in depression, anxiety, and stress. This coincides with other studies indicating that adolescent girls have more mental health problems than their male peers (Fernández-Aramendi et al., 2021). In the case of psychological flexibility and inflexibility, significant differences were also found. Specifically, girls showed greater inflexibility and less flexibility than their counterparts of the opposite sex. This indicates that the psychological rigidity of adolescent girls is higher than that of boys (Livheim et al., 2016). This translates into a cognitive pattern that hinders change and promotes maintaining maladaptive actions (such as experiential avoidance or cognitive fusion). Other studies, such as that of Soares et al. (2023) point in this direction, finding that adolescents had significantly higher scores on psychological flexibility. These authors found that this variable was negatively related to psychological inflexibility and negative affective states (anxiety, depression, and stress). And positively with quality of life and mindfulness. Thus, young people with more flexibility are more focused on the present, have a higher subjective level of health and wellbeing, while they are less constrained by their thinking, since they do not avoid it or try to control it, thus reporting less experience of negative emotional states.

In other words, the greater the individual’s ability to manifest adaptive behaviors, the greater his or her ability to to manifest adaptive behaviors, the greater the capacity to be in the present moment with awareness and acceptance, and the greater the subjective perception of well-being and health. In turn, the greater the the greater the psychological flexibility, the lower the experience of entanglement with thoughts and entanglement with thoughts and consequent avoidance or control of undesirable or control of undesirable internal events, and the lower the experience of negative emotional states.

In terms of mediation analyses, we observed that cybervictimization influences emotional symptoms, through flexibility and psychological inflexibility. The first mediator reduces symptoms of depression, anxiety, and stress linked to cybervictimization. These results are in line with the role assigned to psychological flexibility in previous victimization studies (Duarte and Pinto-Gouveia, 2016; Zhao et al., 2022). However, to our knowledge, this is the first work that points to its mediating role between cybervictimization and emotional symptoms. Adolescents who are victims of cyberaggression would present less psychological flexibility and therefore more difficulties in mastering their behavior and orienting it toward the goals they wish to achieve, which would affect their ability to buffer negative psychological effects.

In the case of psychological inflexibility, this variable acted as a positive mediator of the relationship between cybervictimization and emotional symptoms. Again, we found work that supports these findings. Specifically, as suggested by Makriyianis et al. (2019), early adversity may facilitate depression, stress, and anxiety through inflexibility. The first of these problems would be fostered by promoting the feeling that one is bad or defective for having negative thoughts. Stress would come from inaction or avoidance to experience negative feelings: psychological inflexibility does not allow one to act to achieve desired values if it means going through an unpleasant thought or emotion. Finally, in the case of anxiety, psychological inflexibility promotes getting stuck in one’s thoughts. And when these thoughts are about the future, they provoke anxiety. Therefore, psychological inflexibility would be linked to emotional distress during the adolescent stage (Fernández-Rodríguez et al., 2022), especially among those who face adverse events. In this regard, Makriyianis et al. (2019) have pointed out that young people with more adverse experiences have a higher risk of negative mental health outcomes. And that one of the mechanisms that explain why these risks are transformed into negative outcomes is psychological inflexibility. Online victimization can be understood as an adverse event. So its presence would increase psychological inflexibility and, in turn, tend to worsen young people’s mental health.

Thus, psychological flexibility and inflexibility, central aspects of emotion and thought management (Hayes et al., 2006), play opposing roles in the relationship between cybervictimization and symptoms of anxiety, stress, and depression. Other studies have found that only psychological inflexibility mediated between adverse events and mental health problems; flexibility did not reduce the likelihood of these (Makriyianis et al., 2019). However, the adverse events assessed in Makriyianis et al.’s (2019) study included in many cases maladjustments in the family environment and not only school bullying problems. These events, when originating in the primary developmental domain, may be particularly negative, and psychological flexibility may not be sufficient to alleviate their association. Whereas in the case of cyberbullying, psychological flexibility would play a relevant role, allowing transcending emotions and orienting behavior toward valuable goals, and accordingly, reducing the emotional symptoms caused by cyberaggression.

Finally, this article highlights the importance of psychological flexibility in adverse situations such as cybervictimization during adolescence. Flexibility allows for the adjustment of emotional, cognitive and behavioral responses, favoring adaptation to contextual demands and living in accordance with one’s own values (Cherry et al., 2021). Working on this construct in the educational setting could favor the coping of young victims of cyberbullying. In this sense, Lappalainen et al. (2021) point out that school interventions aimed at increasing psychological flexibility promote mental health among adolescents, since they improve depressive symptoms, life satisfaction, and reduce experiential avoidance. Similarly, Rodríguez-Álvarez et al. (2021) suggest that prevention and intervention in cyberbullying involves working on children’s emotional self-management skills. In addition, adolescence and pre-adolescence would be the optimal time to work on the ability to be flexible and reflective. Advances linked to theory of mind at this stage focus on the development of thinking and emotions linked to themselves. Such as their own perception as psychological and reflective entities, being able to appreciate their private and unconscious worlds (Bialecka-Pikul et al., 2021). Therefore, if psychological flexibility (as a state of mind oriented toward the acceptance of experienced thoughts and emotions; Hayes et al., 2011) is stimulated at this stage of major metacognitive advances, the adolescent would have effective cognitive tools to deal with an increasingly complex social world, as well as with the inherent personal changes of this stage. The elimination of cyberbullying requires education and prevention from an early age. Schools, responsible for the comprehensive education of students, are the ideal place to work on personal skills that protect against the development of these behaviors. And on a second level, schools must act against situations of bullying that arise among their members. Specifically, schools have a particularly relevant role to play in the protection of the victim. Promoting the mental health of young people should be a priority and, to this end, one of the main actions is the implementation of school programs that reduce the risk to mental health and increase the resilience of adolescents (World Health Organization, 2022). Based on the findings of this work, stimulating the psychological resilience of adolescent victims of cyberbullying will reduce emotional symptoms. And finally, to reduce the emotional distress of these young people. Moreover, psychological inflexibility makes it difficult to act toward the achievement of relevant life goals. Therefore, increasing psychological flexibility could lead cyber-victims away from behavior motivated by negative emotions and thoughts, which generates further distress and thus reinforces maladjusted developmental patterns.

### 4.1 Limitations and future investigations

The limitations of this study are related to its cross-sectional design, which does not allow us to establish causal relationships between the variables. On the other hand, the sample consisted of young people aged between 11 and 18 years, and this factor may be differentially linked to the different variables. In future studies, it would be of interest to investigate the possible implication of age in these relationships. Another limitation of this study is that no specific analyses were carried out to ensure item invariance between the groups compared (boys and girls). Invariance is crucial to ensure that observed differences in results reflect real differences in the variables measured, and not differences in the functioning of the measurement instruments. Future studies should incorporate analyses such as Differential Item Functioning (DIF) or multi-group factor invariance, following methodological recommendations such as Hagquist and Andrich (2017), to strengthen the validity of between-group comparisons.

Finally, in the future, it would be of interest to continue investigating the role of flexibility and psychological inflexibility in cyberbullying, from a broader perspective and of particular interest among the scientific community: the transition from cybervictimization to cyberaggression. The scientific literature has pointed out the high association between cyberbullying and being a cybervictim in adolescence, which is a serious public health problem (Brosowaki et al., 2018). Victims may become perpetrators of cyberbullying, as they learn cyberbullying behaviors from their experience of victimization, and are driven by revenge and poor self-control. Moreover, depressive symptoms among victims have been established as a risk factor for cyberbullying perpetration (Tian et al., 2022). From this perspective, just as flexibility and inflexibility mediate the emotional consequences of cyberbullying, attenuating or increasing them, respectively, they could perhaps exert a similar effect on the negative behaviors associated with victimization, such as bullying others. On the other hand, new perspectives on moral development in adolescence are beginning to investigate the effects of technology use on ethical behavior (Malti et al., 2021). Understanding how internal psychological characteristics, such as psychological flexibility, affect moral decisions and behaviors in online social relationships would generate new insights in the field of moral development.

## 5 Conclusion

The introduction of information and communication technologies in the daily life of adolescents has allowed the emergence of cyberbullying in secondary schools. The results of this work show that being a victim of cyberbullying is related to stress, depression and anxiety. And that this relationship is mediated by psychological flexibility and inflexibility. Therefore, the negative psychological outcomes linked to cyberbullying could be reduced through the adolescent’s ability to accept the unpleasant emotions and thoughts resulting from cyberbullying. It is not about changing the adolescent’s thoughts or feelings, but the adolescent’s relationship to them.

These results highlight the importance of the ability to accept one’s own thoughts and emotions, even if they are negative, in problematic situations such as cyberbullying, but they do not reduce the need for families and schools to work together following other strategies focused on promoting social support for the child or education in values for bullies. All of this, together, is beneficial not only for intervening in cyberbullying situations, but also for promoting the adjusted development and psychosocial wellbeing of pupils.

## Data Availability

The data supporting the findings of this study are available from the corresponding author upon request.
